# Osteoinduction and survival of osteoblasts and bone-marrow stromal cells in 3D biphasic calcium phosphate scaffolds under static and dynamic culture conditions

**DOI:** 10.1111/j.1582-4934.2012.01545.x

**Published:** 2012-09-26

**Authors:** Subha N Rath, Leonie A Strobel, Andreas Arkudas, Justus P Beier, Anne-Kathrin Maier, Peter Greil, Raymund E Horch, Ulrich Kneser

**Affiliations:** a Department of Plastic and Hand Surgery, University Hospital of Erlangen Friedrich-Alexander-University of Erlangen-NürnbergErlangen, Germany; b Department of Materials Science (Glass and Ceramics), Friedrich-Alexander-University of Erlangen-NürnbergErlangen, Germany

**Keywords:** osteoblasts, bone-marrow stromal cells, bioreactor, biphasic calcium phosphate (BCP), 3D printing method

## Abstract

In many tissue engineering approaches, the basic difference between *in vitro* and *in vivo* conditions for cells within three-dimensional (3D) constructs is the nutrition flow dynamics. To achieve comparable results *in vitro*, bioreactors are advised for improved cell survival, as they are able to provide a controlled flow through the scaffold. We hypothesize that a bioreactor would enhance long-term differentiation conditions of osteogenic cells in 3D scaffolds. To achieve this either primary rat osteoblasts or bone marrow stromal cells (BMSC) were implanted on uniform-sized biphasic calcium phosphate (BCP) scaffolds produced by a 3D printing method. Three types of culture conditions were applied: static culture without osteoinduction (Group A); static culture with osteoinduction (Group B); dynamic culture with osteoinduction (Group C). After 3 and 6 weeks, the scaffolds were analysed by alkaline phosphatase (ALP), dsDNA amount, SEM, fluorescent labelled live-dead assay, and real-time RT-PCR in addition to weekly alamarBlue assays. With osteoinduction, increased ALP values and calcium deposition are observed; however, under static conditions, a significant decrease in the cell number on the biomaterial is observed. Interestingly, the bioreactor system not only reversed the decreased cell numbers but also increased their differentiation potential. We conclude from this study that a continuous flow bioreactor not only preserves the number of osteogenic cells but also keeps their differentiation ability in balance providing a suitable cell-seeded scaffold product for applications in regenerative medicine.

## Introduction

Bone tissue engineering is intended to treat a bone defect by application of a suitable biomaterial along with osteo-inducing factors or osteogenic cells. A biomaterial for this purpose needs to possess not only appropriate porosity for the ingrowing tissues to invade but also requires suitable mechanical properties for optimal tissue development [1, 2]. A number of scaffold fabrication methods are advocated to produce such porous scaffolds. They are mostly dependent on two methods of fabrication. Firstly, the scaffold can be made with void spaces that are already initially present. This can be achieved, for instance, by rapid prototyping technologies. Secondly, space-filling agents that can later be removed are utilized for generation of porous scaffolds. The so called salt leaching method is one of the most common methods for production of porous implants that is based on space-filling agents [3]. A novel method, combining the above-mentioned processes, allows generation of 3D scaffolds with controlled porosity and architecture. This method relies on 3D printing of biomaterials along with a pore-forming agent such as starch, followed by sintering of the embedded starch to generate the void spaces in the scaffold. After implementation of low-temperature processes, this technology might allow spatially controlled application of growth factors and even direct printing of cells within 3D hydrogel structures [4]. Most of the established scaffold fabrication techniques mix the components thoroughly, so that they are distributed uniformly. In contrast, biological tissues are usually composed of a differentiated architecture based on different cell layers and spatially defined protein composition. Therefore, the uniformity of currently established fabrication methods could not mimic the non-uniform architecture of the defect tissues.

A porous scaffold can functionally be useful only when the media (*in vitro*) or biological fluid (*in vivo*) can freely pass through the scaffold. In addition, cell migration into the central regions of the scaffold as well as cell multiplication is mandatory. The porous network of a scaffold can be functionally assessed *in vitro* in a bioreactor perfusion-culture setting, where the media flowing through it, can be valuable for long-term tissue generation and maintenance of the differentiated tissue phenotype [5]. Although static culture conditions facilitated cell multiplication in 3D constructs in many experimental settings, bioreactors allow for fluid exchange, mechanical load and support cell–cell interaction [6]. They might therefore provide promising conditions for generation of larger volumes of differentiated tissues *in vitro*. Before *in vivo* application of a scaffold, the preliminary testing should be performed in a perfusion-culture system, which allows the provision of a continuous medium transport to the growing tissue, similar to *in vivo* situation [7]. Bioreactor systems have been reported to be useful for differentiation of cells in renal tubular epithelium and skin [7, 8]. In addition, dynamic culture is helpful for increased cell survival and cell proliferation in bone tissue engineering approaches [9]. Therefore, a bioreactor setting is advocated in 3D scaffolds, as it provides conditions that are close to an *in vivo* setting, where the nutrition is supplied *via* dynamic flow processes.

For the development of a bone-like matrix, two types of cells are commonly in use: osteoblasts (OB) and bone-marrow derived stromal cells (BMSCs). OB are differentiated cells, which can progressively deposit calcium matrix to form an osseous product. Different studies have shown their osteogenetic properties, both *in vitro* and *in vivo* [10–12]. On the contrary, BMSCs have significant proliferative capacity and the ability to differentiate into different lineages. These cells have therefore been used in many different bone tissue engineering approaches. Under certain conditions, BMSCs are demonstrated to be osteogenic on biomaterials without any additional factors and grow multiplicatively for a longer passage number than differentiated cells [13, 14]. Osteoblast differentiation can be triggered by mechanical stimuli, which are transduced by osteocytes, which in turn can be stimulated through streaming potentials by fluid flow strain in the lacuna-canalicular system [15].

The purpose of this study is to test whether a continuous-flow bioreactor would provide better culture conditions for induction of long-term differentiation of osteogenic cells (OBs and BMSCs) on a novel 3D printed biphasic calcium phosphate scaffold than a static culture setting. Initially, the scaffold was tested *in vitro* for its biocompatibility using primary OBs. Then dynamic culture conditions in a bioreactor setting were applied to test whether there is any difference in cell growth and differentiation in comparison to static 3D culture under influence of an established dexamethasone-based differentiation medium. Eventually, a comparative study was carried out between primary OBs and BMSCs with regard to osteogenic differentiation capabilities on the scaffold.

## Materials and methods

### Scaffold fabrication

Biphasic calcium phosphate (BCP) scaffolds were fabricated using a three-dimensional printing (3DP) method from three different starting materials: hydroxyapatite (HA; Chemische Fabrik Budenheim, Budenheim, Germany), β-tricalcium phosphate (TCP; Chemische Fabrik Budenheim) and acid-hydrolytic modified potato starch powders (Dextrin; Südstärke GmbH, Schrobenhausen, Germany) in a ratio of 35%-35%-30%, by weight respectively. The details of the fabrication method are described elsewhere (manuscript under preparation). Briefly, cylindrical samples with 10-mm diameter and 5-mm height were 3D printed using a Z-printer 310 (Z Corporation, Burlington, MA, USA). Subsequently, the samples were sintered progressively until 1200°C in an electrically heated furnace in the air to remove the binder to generate the final BCP scaffolds with a three-dimensional interconnected pore system. After cooling, they were ground to dimensions of 5 × 5 × 3 mm^3^. The scaffolds were rinsed thoroughly with simulated body fluid to remove further all non-reacted and non-adhering particles. Samples were then sterilized in 70% ethanol overnight followed by UV light illumination for 4 hrs. Further, they were coated with 0.01% collagen (Sigma-Aldrich, Steinheim, Germany) and washed for 4 hrs in culture media before seeding.

### Material evaluation

For accurate determination of the pores, two different methods in their corresponding range of resolution were used in combination. The porosity with pores less than 52 μm was measured by Hg-porosimetry (Pascal 140; Thermo Electron, Rodano/Milan, Italy). Pore sizes larger or equal to 52 μm were determined by high resolution micro-X-ray computed tomography (μ-CT) analysis (Skyscan 1172; Skyscan, Kontich, Belgium). Samples were scanned at 80 kV voltage, 100 μA current and 5.2 μm voxel size. Data were quantified by a CT reconstruction software (NRecon Client und Serve; Skyscan). The 3D porosity and pore size distributions were calculated by analysis software (CTan 1.10.0; Skyscan). The 3D pore structure was visualized using imaging software (Amira 5.3; Visage Imaging, Berlin, Germany).

The perfusion velocity *v*_perf_ of the medium through the bioreactor scaffolds was calculated according to equation 1, where *Q*_M_ denotes the medium mass flow that perfuses the porous sample (2 ml/hr), and *A* is the area of the sample opposed to the flow rate. As the medium velocity must be regarded with respect to the pores in the scaffold, *A* is multiplied with the porosity *P*_sc_ of the scaffold.


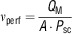
(1)

The flow in the bioreactor was verified by calculating the Reynold's number (Re), Eq. 2. The characteristic path length of a pore was *l*. The fluid kinematic viscosity (*ν*_M_) was determined from the dynamic viscosity of the medium, which was measured by means of a rheometer (Physica UDS 200; A. Paar GmbH, Graz, Austria) using the plate geometry at room temperature.


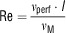
(2)

Compressive strength of the scaffolds (cuboid specimens of 5 × 5 × 3 mm^3^) was measured using a universal testing device (Instron 4204; Instron Corp., Canton/MA, USA) at a crosshead strain rate of 0.5 mm/min. Compressive strength was averaged from the failure stresses of 20 samples.

### Cell isolation

#### Osteoblast isolation and culture

Primary OB were isolated from long bones of male Lewis rats as described elsewhere [16]. In brief, after killing the rats at 8 weeks, the long bones were collected and serially digested in sterile collagenase-II (554 U/ml; Biochrom AG, Berlin, Germany) in 1× PBS. The supernatant of the first digestion step was discarded to prevent any contamination with fibroblasts and BMSCs. The basal culture medium was used as DMEM/ Ham's F-12 (1:1) with 10% FBS, 1% penicillin-streptomycin, 2 mg/l of l-glutamine (all purchased from Biochrom AG, Berlin, Germany). Subsequently, the cells were cultured in flasks (COSTAR, Cambridge, USA), in an incubator with a humidified atmosphere maintained at 37°C and 5% CO_2_. The media were changed twice weekly. At 80–90% confluency, cells were trypsinized (Trypsin/ EDTA; PAA, Pasching, Austria) and cultured further.

#### Bone-marrow stromal cells (BMSC) isolation and culture

The BMSCs from rat bone marrow were isolated according to protocol based on plastic adherence as described earlier [17]. Briefly, femora and tibiae of Lewis rats were collected and the bone marrow plugs were hydrostatically expelled and disaggregated with a needle and a syringe. The medium containing the BMSCs and haematopoietic cells was directly plated in culture plastic and grown until confluency. Seeding results mainly in the simultaneous growth of BMSCs with haematopoietic stem cells [17]. The medium was completely replaced at day 3 after thorough washing by 1× PBS to remove all unattached haematopoietic cells. Thereafter, the medium was changed twice weekly, until the cells achieved 90% confluency. At that point, they were trypsinized and cultured further. After passage 5, the cells were induced to differentiate into three different lineages namely, osteogenic, adipogenic and chondrogenic induction (data not shown).

### Experimental design

The experiment was divided into two groups, as per the cells used, namely group 1 for osteoblast-seeded scaffolds and group 2 for BMSC-seeded ones (Table 1). Each group was further divided into three sub-groups: group A [static culture without osteoinduction (OI)]; group B (static culture with OI started and continued only from week-2); group C (dynamic culture in a bioreactor with OI similar to group B). Samples were evaluated after 3 and 6 weeks. Only second passage cells were seeded onto the scaffolds in group 1 (OB). In the group 2 (BMSC), passage five cells were used.

**Table 1:** Groups and study design. *In vitro* tests are performed with 22 scaffolds per time in group 1 and 14 scaffolds per time in group 2 (3 and 6 weeks). Primary osteoblast (Group 1) and bone marrow stromal cell (Group 2) seeded scaffolds were cultured in static conditions (Group A), static with osteoinduction (OI) media (Group B), dynamic culture in bioreactor with OI (Group C)

Cell groups	Groups (*n*= )	Culture conditions	Alamar blue	ds-DNA	ALP assay	Live-dead assay	SEM	RT-PCR	Time points
1 (OB)	A	Static	(22)	8	(8)	2	2	10	Week 3, Week 6
	B	Static with osteoinduction	(22)	8	(8)	2	2	10	Week 3, Week 6
	C	Dynamic with osteoinduction	(22)	8	(8)	2	2	10	Week 3, Week 6
2 (BMSC)	A	Static	(14)	4	–	1	1	10	Week 3, Week 6
	B	Static with osteoinduction	(14)	4	–	–	–	10	Week 3, Week 6
	C	Dynamic with osteoinduction	(14)	4	—	—	—	10	Week 3, Week 6

ALP: alkaline phosphatase; BMSC: bone marrow stromal cells; OB: osteoblasts.

All scaffolds were seeded with 8 × 10^4^ cells per scaffold. The scaffolds used for dynamic culture conditions are described later. The static-cultured ones were supplied either with basal culture media or with differentiation media from week-2 in the incubator as used for cell culture. The differentiation medium was composed of basal culture medium with added 0.1 μM dexamethasone, 50 μg/ml of ascorbic acid, 10 mM β-glycerophosphate (all from Biochrom AG, Berlin, Germany). At the end, all scaffolds were analysed as per Table 1.

### Bioreactor experimental set-up

The cell-seeded scaffolds from groups 1C (OB) and 2C (BMSC) were set-up in a perfusion chamber (Number 1301; Minucells and Minutissue, Bad Abbach, Germany) 3 days after static culture, as described originally before [18]. Briefly, eight cell-seeded scaffolds were put per perfusion chamber, which was placed onto a 37°C heating plate (Medax, Kiel, Germany). The scaffolds were cultured either for 3 weeks or 6 weeks, of which the first week was in basal media, followed later by differentiation media; all were buffered by additional 1% Bufferall (Sigma-Aldrich GmbH). The medium was continuously perfused at a flow rate of 2 ml/hr with an IPC N16 peristaltic pump (ISMATEC, Wertheim, Germany) which allowed a continuous exchange of media. The flow rate was chosen based on preliminary experiments that demonstrated optimal cell attachment and differentiation (data not shown).

### Evaluation techniques

#### AlamarBlue assay

The cell-seeded scaffolds were analysed by alamarBlue (Biosource Int., Camarillo, CA, USA) assay. Each week, the scaffolds from groups 1 A/B and 2 A/B were washed with 1× PBS and 500 μl of the culture medium with 10% alamarBlue was added and incubated for 3 hrs at 37°C. Absorbance was then measured with a plate reader (SPECTRAmax 190; Molecular Devices, Sunnyvale, CA, USA) at wavelengths of 570 nm and 600 nm. The percentage of alamarBlue reduction was subsequently calculated as advised by the manufacturer from the absorbance values of cell-seeded scaffolds, alamarBlue mixture, and culture medium. In the bioreactor groups, 1C and 2C alamarBlue assays were performed as end-point analysis at weeks 3 and 6 as described above.

#### Picogreen and ALP assay

To analyse the cell proliferation and ALP content, the cell-seeded scaffolds were washed with 1× PBS and then treated by lysis buffer (10 mM Tris -pH 7.0, 1 mM EDTA, and 0.2% v/v triton X-100; all from Sigma-Aldrich GmbH). The lysates were frozen at −80°C and later analysed. Cell proliferation was evaluated by measuring dsDNA using PicoGreen DNA quantification assay (Molecular Probes; Invitrogen GmbH, Karlsruhe, Germany) at 3 and 6 weeks, according to the manufacturer's protocols. The fluorescence was measured at excitation and emission wavelength of 485 nm and 520 nm respectively (Genios; Tecan Group Ltd, Maennedorf, Switzerland).

The ALP content was assessed by the measurement of the coloured complex produced by the hydrolysis of para-nitrophenyl phosphate (p-NPP) (Sigma-Aldrich), as advised by the manufacturer. The thawed lysate was incubated with substrate mixture consisting of diluted p-NPP in the alkaline buffer at 37°C for 30 min. The reaction was terminated by adding diluted sodium hydroxide. The developed colour was measured by the absorbance at 405 nm using lysate buffer as blank by a plate reader (SPECTRAmax 190; Molecular Devices). ALP activity values were calculated after applying suitable correction factors from a standard calibration curve. The values were normalized to dsDNA content.

#### FDA/PI staining for live-dead assay

The cell-seeded scaffolds were washed with 1× PBS and incubated with 2 μg/ml fluorescein diacetate (FDA) (Molecular Probes Inc., Eugene, US) in 1× PBS, for 15 min. at 37°C. They were then gently rinsed twice in 1× PBS and placed in 20 μg/ml propidium iodide (PI) solution (Invitrogen GmbH) for 2 min. at room temperature. After thorough rinsing in 1× PBS, the specimens were kept in PBS and viewed under a fluorescent microscope (Axiovert 25; Carl-Zeiss AG, Goettingen, Germany). The viable-cell cytoplasms were labeled green, while non-viable cell nuclei were labeled red.

#### Scanning electron microscopic (SEM) analysis

The cell-seeded scaffolds were washed with 1× PBS and fixed in 2.5% glutaraldehyde for 1 hr and freeze-dried. Subsequently, both their surface and their interior parts were analysed microscopically by a SEM (Quanta 200 instrument; FEI, Prague, Czech Republic).

#### Real time PCR analysis

Total RNA was isolated from the cell-seeded scaffolds (*n* = 10) using TRIzol Reagent (Invitrogen, Carlsbad, CA, USA) followed by RNeasy Mini Kit (Qiagen, Hilden, Germany) as per the manufacturer's recommendation with few modifications [19]. Total RNA was converted to cDNA (QuantiTect reverse transcription kit; Qiagen). The amount of cDNA corresponding to 20 ng of total RNA was then analysed in duplicates as eight independent measurements by semi-quantitative real-time PCR (iQ SYBR green; Bio-Rad, Munich, Germany) for selected genes with primers as shown in Table 2 (CFX 96 real time systems; Bio-Rad, Munich, Germany). The gene expressions were normalized to internal GAPDH expression, and the relative fold change was expressed by comparing to that of control group 1A for OB and to 2A for MSC groups.

**Table 2:** The primers used in real time RT-PCR analysis

Gene	Forward primer	Reverse primer
GAPDH	TGGCCTCCAAGGAGTAAGAA	TGTGAGGGAGATGCTCAGTG
RUNX2	CCACCACTCACTACCACACG	TATGGAGTGCTGCTGGTCTG
ALP	GCTGATCACTCCCACGTTTT	GCTGTGAAGGGCTTCTTGTC
Osteocalcin	CATGAGGACCCTCTCTCTGC	TTCACCACCTTACTGCCCTC
Osteopontin	GATCGATAGTGCCGAGAAGC	TGAAACTCGTGGCTCTGATG
Osteonectin	GAGGCCATAGCCTATCCACA	AGGAAGGCAAGCTTATGCAA
IBSP	GAAGCAGGTGCAGAAGGAAC	GAAACCCGTTCAGAAGGACA

IBSP: integrin-binding sialoprotein.

#### Statistical analysis

Statistical comparisons were performed for histomorphometric analysis using a two-way anova test followed by Bonferroni's post-test (Sigmastat v3.5, Chicago, IL, USA) considering a significant difference at the 95% confidence interval. Standard deviations were included in all graphs and texts at *P* = 0.05 or 0.001 level as mentioned. For all pairwise comparisons on quantitative results, the Student's *t*-test was used with a confidence level of 95% (*P* < 0.05).

## Results

### Scaffold properties

**Fig 1:**
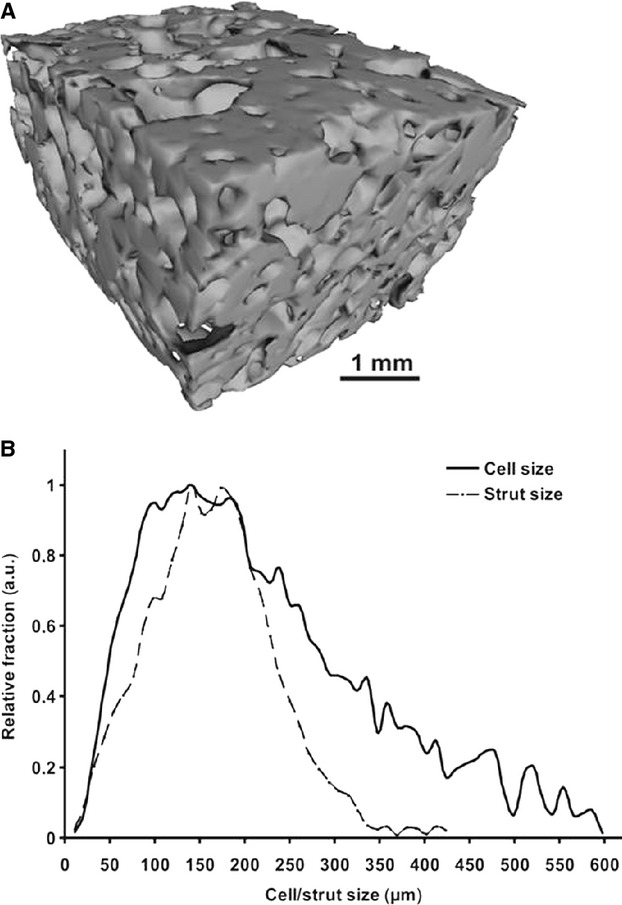
Physical properties of the scaffold: (A) Micro CT reconstructed image showing the gross picture and (B) the analysed pore distribution. The porosity is 65.3%.

The BCP scaffolds exhibit a porous 3D interconnected pore structure with non-uniformly arranged pores. Visual observation and micro-CT reconstructed images demonstrate their porous architecture with pores up to 600 μm size (Fig. 1). The total porosity calculated from Hg-porosimetry and μ-CT measurements is 65.3%. The compressive strength of the scaffolds is 3.4 ± 0.6 MPa. The perfusion velocity of the culture medium through the 3D pore system is 2.1 mm/s. The calculated Reynold's number is 0.22 (for characteristic path length *l* = 100 μm), which ensures a laminar flow during perfusion experiments.

### Primary osteoblast-seeded constructs (group 1)

**Fig 2:**
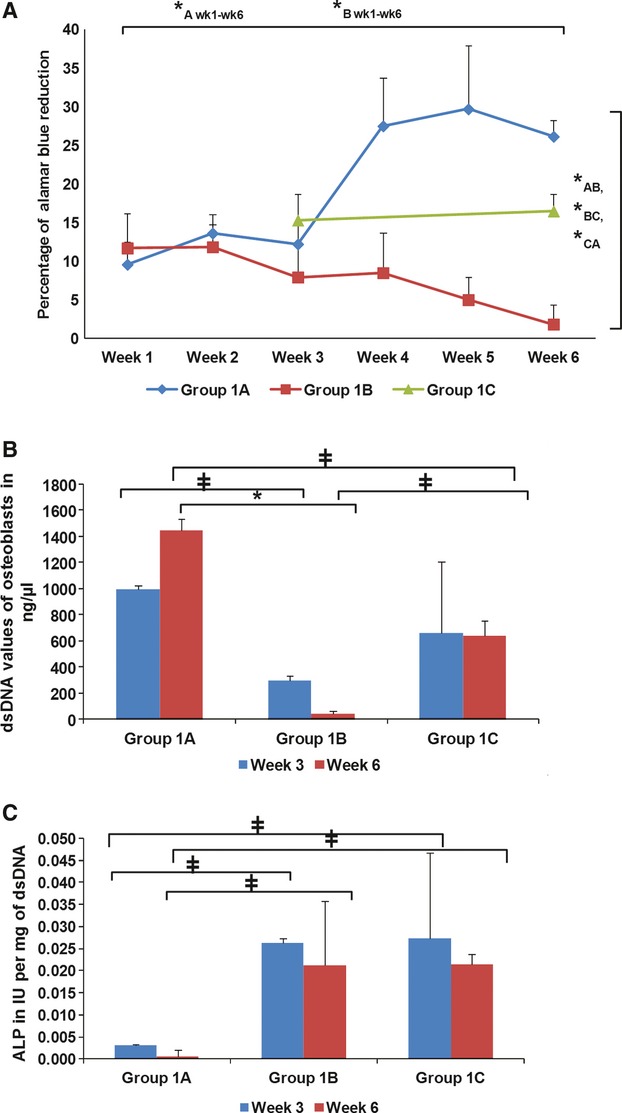
*In vitro* analysis of osteoblast-loaded constructs (group 1). (A) AlamarBlue assay for 6 weeks. (B) DNA quantification assay. (C) Alkaline phosphatase values standardized per mg of dsDNA. Group 1A = static culture; group 1B = static culture and osteoinductive (OI) media; group 1C = dynamic culture and OI media. The statistical significance level is indicated by *at *P* = 0.001 level and at *P* = 0.05 level between the mentioned groups.

The metabolic activity of the osteoblast-seeded constructs (group 1) in all culture conditions remains similar until 3 weeks, as seen by alamarBlue dye reduction assay (Fig. 2A). Thereafter, the metabolic activity of the constructs in group 1A (static culture without OI) increases progressively, whereas that in group 1B (static culture with OI) declines over 6 weeks. The values at week-6 in groups 1A and 1B are significantly different from those at week-1 (*P* = 0.001). In contrast, the constructs in dynamic culture even with osteoinduction (group 1C) show a persistently similar dye reduction without any significant difference. At 6 weeks group 1A displayed the highest alamarBlue reduction, whereas the reduction in group 1C was still higher than in group 1B (*P* = 0.001).

The dsDNA quantification assay values reflecting the number of cells in the constructs correspond well with the alamarBlue assay results (Fig. 2B). The number of cells is highest in group 1A, especially at week-6 compared with the values of groups 1B (*P* = 0.001) and 1C (*P* = 0.05). A significantly lower dsDNA content is observed at week-6 in group 1B compared with group 1C, although both are in differentiation media (*P* = 0.05).

The ALP values standardized to mg of dsDNA indicate that samples from both groups 1B and 1C show significantly higher values than the non-induced static-cultured group 1A constructs at both time points (*P* = 0.05). There is no significant difference between groups 1B and 1C at any time point (Fig. 2C).

**Fig 3:**
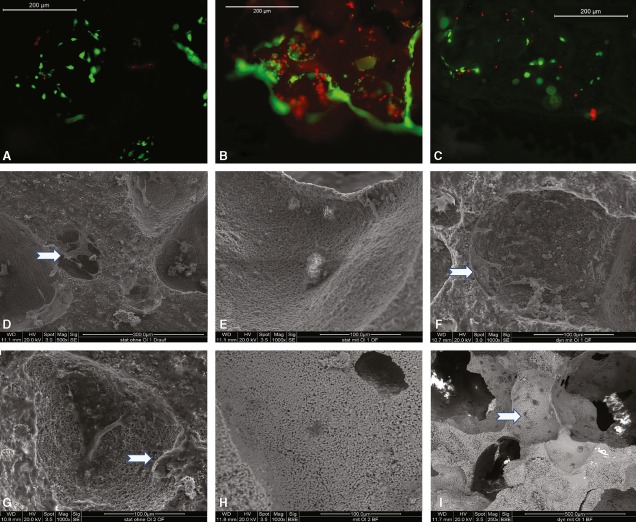
*In vitro* analysis of osteoblast-loaded constructs (group 1). (A–C) Fluorescent microscopic observation of live cells (green) and dead cells (red) (A) Group 1A, (B) Group 1B, (C) Group 1C. (D–I) Scanning electron microscopic images of the constructs: (D–F) Week 3, (G–I) Week 6. (D,G) Group 1A, (E,H) Group 1B, (F,I) Group 1C. Please note the different scale bars. The scaffold interiors are examined in (H, I). Arrows indicate the cells. Details are described in the text.

Vitality of OB is demonstrated at week-6 by FDA/PI staining (Fig. 3A–C). Although the number of cells is observed to be comparable in all samples, the viable cell numbers differ. In constructs from group 1A, most of the cells are alive with only a limited number of dead cells. In contrast, in samples from group 1B many of the embedded cells are dead, as depicted by red PI-stained cells. A comparable number of dead and live cells as in group 1A are observed in group 1C (dynamic with OI) samples, where most of the cells are alive. In all groups, the cells are found around the pores deep in the scaffolds.

In SEM analysis, group 1A scaffolds show numerous cells, which spread well with many pseudopodia-like processes forming a cell-sheet like morphology by 3 weeks. By that time, group 1B scaffolds show minimum number of attached cells, whereas group 1C scaffolds possess numerous cells even deep in the pores of the scaffolds (Fig. 3D–F).

By 6 weeks, group 1A constructs still show numerous cells, which are uniformly distributed all over the scaffold surface (Fig. 3G). Group 1B constructs show few cells in the scaffold interior when examined inside a broken scaffold (Fig. 3H). In contrast, group 1C constructs show numerous well-spread cells in the interior (Fig. 3I) in addition to their finding on the surface, which is remarkable and specific only for this group.

**Fig 4:**
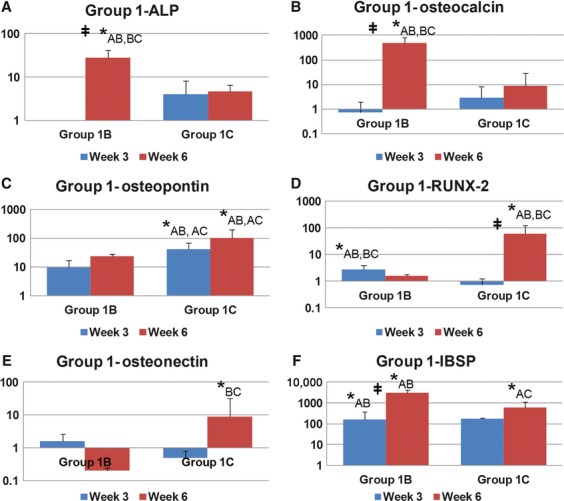
Quantitative real time RT-PCR analysis of bone related gene expression in osteoblast (OB)-loaded constructs (group 1). (A) alkaline phosphatase, (B) osteocalcin, (C) osteopontin, (D) RUNX-2, (E) osteonectin, and (F) integrin-binding sialoprotein (IBSP). Specific gene expression was normalized to internal GAPDH expression. Values represent the fold change compared with control group A of respective time points. Each bar represents 8 independent measurements. The statistical significance at *P* = 0.05 is indicated either by *between groups at same time or between time points of same group. Group 1A = scaffolds in static culture without OI, group 1B = scaffolds in static culture with OI, group 1C = scaffolds in dynamic culture with OI.

Bone-specific gene expression profile for osteoblast-seeded scaffolds is shown in Figure 4. The results are expressed as fold change of their expression with respect to group 1A (static without OI) samples at corresponding time points, normalized to their GAPDH expression. The relative expressions of ALP and osteocalcin are significantly increased in group 1B constructs, whereas those of osteopontin, osteonectin and RUNX2 are significantly increased in group 1C ones, compared with control group 1A. This difference is most evident at 6 weeks. The relative expressions of integrin-binding sialoprotein (IBSP) are significantly increased in both groups. Collagen-1 expression was significantly decreased under the influence of the dexamethasone-based differentiation media (data not shown).

### Bone-marrow stromal cell-seeded constructs (group 2)

**Fig 5:**
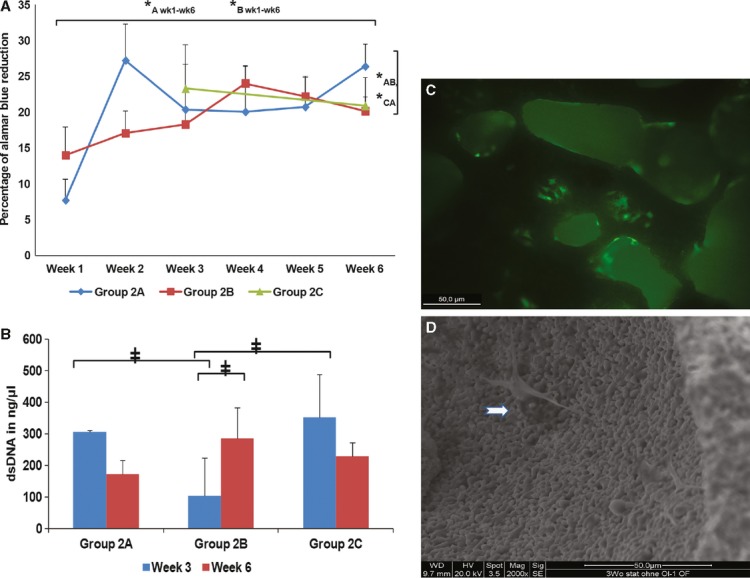
*In vitro* analysis of BMSC-loaded constructs (group 2). (A) AlamarBlue assay for 6 weeks. (B) DNA quantification assay. (C) Fluorescent microscopy for live cells (green) and dead cells (red) (group 2A static culture without OI); (D) scanning electron microscopy for cell adhesion and spreading ) (group 2A static culture without OI). For details refer text. OI = osteoinduction. Group 2A = static culture; group 2B = static culture with OI media; group 2C = dynamic culture with OI media.

Over 5 weeks, there is a comparable metabolic activity of BMSC-seeded constructs as seen by alamarBlue dye reduction assay in all three different culture systems, basically independent of osteoinduction (Fig. 5A). Both the statically cultured groups (2A static without osteoinduction, 2B static with osteoinduction) show increased dye reduction at week-6 compared with week-1 (*P* = 0.001). At 6 weeks time, it is observed that constructs with osteoinduction (group 2B static with osteoinduction, group 2C dynamic with osteoinduction) have significantly decreased values compared with those without osteoinduction, irrespective of their culture conditions (*P* = 0.001). However, when compared with osteoblast-seeded constructs (group 1), the BMSC-seeded scaffolds show increased magnitude of dye reduction.

The dsDNA values of BMSC-seeded constructs show that the values of groups 2A (static without osteoinduction) and 2C (dynamic with osteoinduction) are significantly increased in comparison with group 2B (static with osteoinduction) at week-3 (*P* = 0.05) (Fig. 5B). However, the values show no significant difference by week-6. In the live-dead assay, the static culture group 2A shows a number of live cells indicating a stable association with the biomaterial (Fig. 5C). In SEM, the static culture group 2A also shows BMSC attachment and spreading on the biomaterial (Fig. 5D).

**Fig 6:**
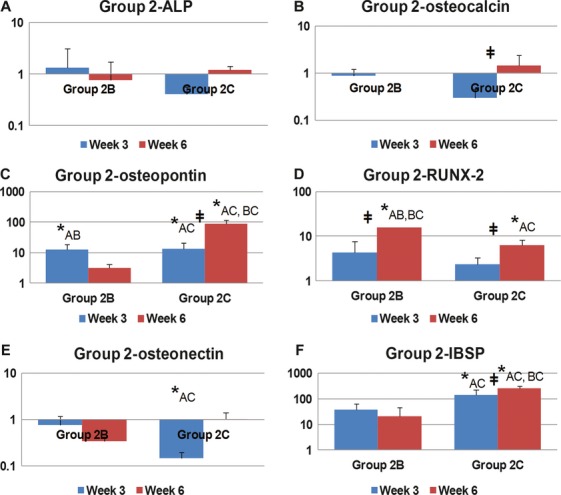
Quantitative real time RT-PCR analysis of bone related gene expression in BMSC-loaded constructs (group 2). (A) alkaline phosphatase, (B) osteocalcin, (C) osteopontin, (D) RUNX2, (E) osteonectin, (F) integrin-binding sialoprotein (IBSP). Specific gene expression was normalized to internal GAPDH expression. Values represent the fold change compared with control group A of respective time points. Each bar represents eight independent measurements. The statistical significance at *P* = 0.05 is indicated either by *between groups at same time or between time points of same group. Group 2A = scaffolds in static culture without OI, group 2B = scaffolds in static culture with OI, group 2C = scaffolds in dynamic culture with OI.

Bone-related gene expression profile for BMSC-seeded scaffolds is shown in Figure 6. The expression levels of ALP, osteocalcin and osteonectin are not significantly increased among the three groups. In contrast, RUNX2 and osteopontin are significantly increased in groups 2B and 2C in comparison with control group 2A at one or both time points; IBSP expression is significantly increased in group 2C compared with group 2A at both time points.

## Discussion

We have been able to demonstrate suitable biocompatibility of the novel 3D-printed BCP scaffolds *in vitro* under basal and osteoinductive culture conditions with different types of osteogenic cells. The dynamic culture in a bioreactor system improved survival and osteogenic differentiation of the seeded cells on the scaffold. Primary OB and BMSCs under dynamic culture conditions showed comparable potential whereas a significant difference was observed in static culture, especially following osteoinductive stimuli. To the authors' knowledge, no previous study has been attempted to compare the *in vitro* osteogenic capabilities of the two most-common osteogenic cells (OB and BMSCs) in 3D printed biphasic ceramic scaffolds.

### Material properties

The compressive strength of the 3D printed porous BCP scaffolds was about 3.5 MPa, which is in the range of normal cancellous bone (2–5 MPa) [20]. The medium flow through the porous BCP scaffolds in the bioreactor perfusion is laminar as indicated by the Reynold's number (Re = 0.22). As long as Re is less than the critical Re value (1–10 for porous materials), a laminar flow of the respective fluid is ensured [21]. The dynamic laminar flow condition has been shown to promote the diffusion of nutrients and metabolites; therefore, it permits the fabrication of viable biological 3D tissue equivalents [22]. The 3D BCP scaffolds eventually maintained satisfactory cell survival and supported differentiation of osteogenic cells as demonstrated by morphological and molecular biological investigation.

### Differentiation and attachment of OBs and BMSCs

Analysis of osteoblast-seeded constructs from group 1 revealed a complex cellular response to individual culture conditions. AlamarBlue and dsDNA quantification assays as well as live-dead assays indicate that a relevant proportion of OB left the proliferation phase and might have become apoptotic after they have been induced for differentiation. This effect might have been caused by the dexamethasone supplement in the differentiation media [23]. As expected, osteogenic differentiation induced OB to synthesize ALP, one of the important markers for bone differentiation. However, osteo-induced constructs from group 1B might not be usable with such low numbers of cells. A clear benefit is visible in the bioreactor constructs, as they not only stabilize enough OB but also differentiate a good fraction of them in the osteogenic lineage as demonstrated by increased ALP activity and osteogenic gene expression. Obviously, the dynamic culture not only improved osteoblast survival in the centre of the scaffolds but also supported cells despite the use of the above-mentioned dexamethasone-added differentiation media. These results are clearly expressed in the live-dead assay, which shows a large number of vital cells within the entire scaffold. A comparable scenario is observed in BMSC-seeded scaffolds. Although the trend is similar, the assumable apoptotic effect of dexamethasone-mediated osteoinduction is smaller. This is further confirmed by the SEM images and live-dead assay of both cell types on the scaffold. Bone morphogenetic proteins (BMP) have been used as alternative osteo-inducing agents, but mainly in *in-vivo* situations. Their *in vitro* ability of osteoinduction is considered similar to dexamethasone; rather, it has been demonstrated that they decreased osteoblast number lower than that in dexamethasone *in vitro* [24]. In future *in vivo* experiments, induction of osteogenic differentiation will be achieved by application of BMP in the porous BCP scaffolds.

Bone formation is regulated through a hierarchical expression of transcription factors. RUNX2 is an essential transcription factor for endochondral bone formation. The real time RT-PCR results show significantly increased expression levels of osteocalcin, ALP and osteopontin under either static or dynamic conditions with osteoinduction. RUNX2 and IBSP expression is increased in all the osteo-induced specimens indicating their differentiated status. IBSP is the extra-cellular matrix protein that binds hydroxyapatite for bone formation [25]. This is significantly increased in all osteo-induced specimens. Osteonectin induces calcification in collagen for bone formation. RUNX2 in BMSCs results in up-regulation of osteoblast-specific genes, including osteocalcin, osteopontin, ALP and collagen type I [15]. The osteogenic gene expression is typically found after 3 weeks of osteoinduction [11].

### Role of bioreactor technology

The number of osteogenic cells, cultivated in perfusion culture, is not increased after osteoinduction; although they show a significantly increased ALP activity [26]. The survival of a sizeable cell-scaffold construct is limited by gradients in oxygen diffusion from the periphery toward the centre. It is still an unsolved challenge to deliver oxygen homogenously in 3D cell cultures. This in turn impedes uniform cellular growth in scaffolds. Within 5 days in static culture, the oxygen concentration in the centre of the construct drops to 0%, causing the central cells to die. The use of perfusion bioreactors successfully prevents cell death, yet central oxygen concentrations do not rise above 4% [27]. *In vitro*, sufficient nutrition and oxygenation of cells by diffusion is limited to a distance of 100–200 μm [27, 28]. As oxygen has poor diffusion capacity and solubility, hypoxia is the limiting factor in scaling up 3D cultures. Recent studies have provided proof that the osteogenic differentiation is also highly dependent on oxygen levels [27]. Our results indicate that static 3D culture might create oxygen gradients, which prevent proper cell survival. In the current study, this effect was intensified by osteoinductive stimuli. With dynamic culture, the oxygen gradient might be alleviated with consecutive higher cell survival, even under osteoinductive conditions. In addition, flow perfusion bioreactor systems might provide direct and indirect mechanical stimulation [27]. The dynamic environment generated by just oscillating the scaffolds has been reported to promote up to 150% higher cellular load within 1 week than static culture [29]. Cell distribution in the scaffold was more homogeneous under dynamic than under static conditions, because of its effect on seeding efficiency and cellular attachment

### Potential application and outlook: optimization of proliferation and differentiation

A markedly decreased cell number has been reported in the osteogenic-induced scaffolds, especially under static culture conditions [30]. This may have multiple causes. Dexamethasone treatment triggers a distinct set of pathways, including apoptosis signalling and calcium signalling [31, 32]. In 3D culture with a dose-dependent effect, it increases the number of apoptotic cells, decreases the replication of osteoblastic cells, as well as enhances bone formation and osteoblastic cell differentiation [23, 33]. *In vivo* apoptotic OB and osteocytes are demonstrated in patients with glucocorticoid-induced osteoporosis [34]. In contrast, there is a protective action of dexamethasone against apoptosis of BMSCs, which occurs at higher cell densities because of direct cell contact [35]. In addition, the roughness of the scaffolds may alter the differentiation ability of BMSCs with larger diameter tubes causing dramatic apoptosis [31, 36]. The current study clearly demonstrates that dynamic culture conditions support cell survival and differentiation under dexamethasone-mediated osteoinduction. This culture setting might therefore be used for optimization of *in vitro* generation of bioartificial bone tissues, if static culture conditions do not facilitate adequate balance between cell proliferation and osteogenic differentiation. However, it has to be noted, that for the comparative analysis of cell survival in dynamic culture with osteoinduction, an additional control group including dynamic flow conditions without osteoinduction would be needed.

The yield of direct isolation of BMSCs is very low, making expansion a required step. It has been shown that after passage 6, BMSCs have reduced capacity to mineralize and to induce ALP by dexamethasone [37]. We have tested their differentiation ability in the form of mineralization ability at each passage before use (data not shown). However, it was reported that they enter senescence and start to lose their stem cell characteristics almost from the very moment the *in vitro* culture begins [38]. These studies and our results suggest that with every passage, there is a definite mixture of cells with high differentiation ability along with senescent cells. Gómez *et. al*. have advocated that, although a number of defined criteria are important to procure BMSCs, they are not sufficient to explain differences in the behaviour of BMSCs obtained from different sources or individuals at present, both *in vitro* and *in vivo* [39]. Therefore, BMSCs should always be carefully implemented for tissue engineering applications.

Although our study shows only a marginal osteogenic advantage of OB over BMSCs, the *in vivo* picture might be different. In a murine *in vivo* model, BMSCs and OBs were reported to have different pathways of bone formation in ceramic porous scaffolds. BMSCs form bone by the endochondral pathway whereas OB induce mainly intramembranous ossification. BMSCs have a potential added advantage *in vivo*, as they recruit host endothelial cells and induce vascularization, which is strongly linked to endochondral ossification [12]. Interestingly, in this study the transplanted BMSCs were not found after 30 days of their *in vivo* application. The authors concluded that the osteocytes in the newly generated bone within the BMSC-seeded scaffolds did not originate from the transplanted BMSCs, but were rather of host origin [12]. It is noteworthy that BMSCs represent a less committed stage of the OB lineage, as they physiologically differentiate to OB during the osteogenic process.

The 3D printing method applied in this study can be very useful in producing specific functional scaffolds. In the future, after implementation of low-temperature processing protocols even joint application of growth factors and / or functional cells might be facilitated. Simultaneous printing of cells and biomaterials has already shown precisely to place cells and proteins within 3D hydrogel structures [40].

## Conclusion

This study demonstrates that the 3D printed BCP scaffolds possess suitable porosity and internal structure for laminar fluid flow inside them. The static culture with osteoinduction had a detrimental effect on cell survival over a prolonged period of time. However, in dynamic culture the same osteoinductive conditions not only favoured osteogenic cell survival but also supported their differentiation and functional outcome in BCP scaffolds. Dynamic culture might therefore be employed for generation of large cell-loaded 3D composites, if osteoinductive dexamethasone-containing cell culture media are used. The study further highlights the difference between OB and BMSCs in 3D culture under osteoinductive conditions. This study justifies further *in vivo* experiments, where the novel 3D printed BCP scaffolds should be explored with a focus on bone tissue engineering applications.
